# An Improved Kernel Credal Classification Algorithm Based on Regularized Mahalanobis Distance: Application to Microarray Data Analysis

**DOI:** 10.1155/2018/7525786

**Published:** 2018-06-27

**Authors:** Khawla EL bendadi, Yissam Lakhdar, El Hassan Sbai

**Affiliations:** ^1^Department of Physics, Faculty of Sciences, Université Moulay Ismail, Meknes, Morocco; ^2^High School of Technology, Université Moulay Ismail, BP 3103 Route d'Agouray, 50006 Toulal, Meknes, Morocco

## Abstract

Within the kernel methods, an improved kernel credal classification algorithm (KCCR) has been proposed. The KCCR algorithm uses the Euclidean distance in the kernel function. In this article, we propose to replace the Euclidean distance in the kernel with a regularized Mahalanobis metric. The Mahalanobis distance takes into account the dispersion of the data and the correlation between the variables. It differs from Euclidean distance in that it considers the variance and correlation of the dataset. The robustness of the method is tested using synthetic data and a benchmark database. Finally, a set of DNA microarray data from Leukemia dataset was used to show the performance of our method on real-world application.

## 1. Introduction

Clustering methods are used to classify samples according to specific properties; they consist of organizing a set of *n* objects *X* = {*x*_1_, *x*_2_,…, *x*_*n*_} into *c* clusters, in order to have a high degree of similarity within these clusters. These approaches are applied in a wide variety of areas including data mining, pattern recognition, and computer vision.

Clustering methods were developed by different ways from which we find the hard, fuzzy, and evidential approaches. In the hard clustering [1], clusters are disjointed and does not overlap; we find many algorithms based on kernels which have developed in [2, 3]. On the other hand, in fuzzy clustering [4, 5], a pattern may belong to a number of clusters with a degree of membership.

However, for evidential clustering [6], the concept of metacluster has been introduced to manage the points belonging to overlapping areas.

The theory of belief functions, also known as Dempster-Shafer theory or evidence theory, is an extension of fuzzy approach. It has been shown to be a powerful framework for representing and reasoning with uncertain and imprecise information. The credal classification works under the framework of this theory also called evidential reasoning [7].

A growing number of applications of belief function theory have been reported in unsupervised learning [6, 8, 9], semisupervised learning [10], image processing [11, 12], social network analysis [13], and data analysis of large dissimilarity data [14, 15].

In the literature, there are several methods based on the concept of evidential reasoning as evidential C-means (ECM) [8], Credal C-means (CCM) [16], and credal classification rule (CCR) [17]. In the CCR approach, each pattern is assigned not only to a single cluster, but also to the metacluster. Two varieties of CCR algorithm are developed, one uses Euclidean distance [17] and another version is improved to take into account the data dispersion [18].

However, most of these clustering algorithms present some limits. These methods use Euclidean distance to calculate the similarity between the centers of clusters and data points in the input space. This similarity performs well with hyperspherical data and may not be significant or even deceptive for categorical data type. However, an appropriate clustering method can be introduced to overcome these limitations when the data structure is complex.

Given these limitations, kernel methods have been proposed. Among these methods which have been applied into many learning systems, we find Support Vectors Machine (SVM) [19], Kernel Fuzzy C-means (KFCM) [20], and Kernel Evidential C-means (KECM) [21]. In the same sense, a kernel version of credal classification rule (KCCR) [22, 23] has been recently proposed. KCCR adopts a new kernel metric in the data space to replace the Euclidian distance in CCR with an appropriate kernel function.

When the dissimilarity measure is based on the Euclidean distance, it only characterizes the mean information of a cluster, and therefore it is sensitive to noise and affects cluster divergence. For this, in this paper, we propose a new clustering algorithm which uses the regularized Mahalanobis distance as dissimilarity in the kernel function. On the one hand, the Mahalanobis distance is a useful measure that determines the similarity between datasets. It takes into account the variance and correlation of the data distribution and is often used to detect aberrant data. On the other hand, the computation of the inverse covariance is perhaps impossible or at least unstable. This problem can be classified as inverse, ill-posed, or ill-conditioned problem. This problem is avoided by different methods such as regularization [24, 25].

The article is organized as follows. In the first section, a compendium of credal classification methodology is described, with the CCR algorithm and its kernel variant. An improved version of KCCR method using Mahalanobis distance is detailed in Section 2. In Section 3, the experimental results using simulated data and many examples from the benchmark database will be presented to show the performance of the method. Also, an application on Leukemia dataset is described in this section in order to better evaluate our contribution. The conclusion is given in Section 4.

## 2. Overview of Credal Classification

The subject of credal classification is exactly to model classification under conditions where not much information is present, by allowing the classifier to produce sets of outcomes rather than single outcomes. The credal classification is based on the theory of belief functions also called evidential reasoning. It allows the objects to belong with different masses of belief, not only to a singleton cluster, but also to a set of clusters named metacluster. The credal classification of the objects is obtained by the combination of the basic belief assignments (BBA) associated with different classes.

In the literature, one can find different approaches, as well as many applications in several fields for the use of credal partition, such as credal C-means (CCM) [16], belief C-means (BCM) [26], and credal classification rule (CCR) [17]. In the next section, brief recall of CCR approach is presented. After that, a kernel credal classification rule (KCCR) is held.

### 2.1. Credal Classification Rule

The credal classification rule works with credal partition; it calculates the masses of beliefs associated with the specific cluster, the metacluster, and outlier cluster in a simple way.

The interest of the credal classification is clearly evident when the information is insufficient to properly classify an object. For this purpose, the CCR algorithm assigns these objects to the metaclusters (cluster of imprecise objects) computed under the framework of the belief functions. In fact, the credal classification can well reduce errors and correctly capture the imprecision of the classification for dealing with the uncertain data.

A basic belief assignment (BBA) is a function *m*(.) from 2^Ω^ to [0,1], where Ω represent the frame of discernment satisfying *m*(*∅*) = 0 and ∑_*A*∈2^Ω^_*m*(*A*) = 1. Two steps are required for the implementation of the CCR approach. First, we start by determining the centers of different cluster types, after we calculate the distance between the objects to be classified and each cluster center in order to construct the BBA.

The computation of the specific cluster center can be established by different methods. As the calculation of the average of the training data or use the centers retrieve by a classification algorithm like FCM or ECM. The determination of the center of the metacluster *c*_*U*_ is considered with the same distance to all the involved centers of the singleton clusters *c*_*i*_.

The mass of object associated with singleton and metacluster (union from at least two specific clusters) is calculated by the distance between the object *x*_*s*_ ∈ *X*, *s* = 1,…, *n* and the corresponding cluster (singleton or meta) centers. The mass of belief on specific cluster and metacluster is given by the following [17]:

(1) m~wi=e−λdβxs,ci

(2) m~U=e−λΔxs,cU

where

(3) $$\begin{array}{c} \Delta \left(\;x_{s},c_{U}\;\right)≜\frac{1}{\left|U\right|+\gamma }\left[\;\underset{w_{i}\in U}{\sum }d^{\beta }\left(\;x_{s},c_{i}\;\right)+\gamma d^{\beta }\left(\;x_{s},c_{U}\;\right)\;\right] \end{array}$$

The parameter *γ* is an adjustment-weighting factor of the distance between the object and the center of metacluster. The tuning parameter *β* can be fixed to a small value (1 or 2).

The mass of belief for the outlier cluster (all objects very far from all clusters) is controlled by the parameter *δ*, according to the following formula:

(4) $$\begin{array}{c} \tilde{m}\left(\;w_{0}\;\right)=e^{-\lambda \delta } \end{array}$$

where

(5) $$\begin{array}{c} \delta =\eta \times \arg\left[\;\max\left(\;d^{\beta }\left(\;c_{i},c_{j}\;\right)\;\right)\;\right] \end{array}$$

In order to overcome the limitations of CCR method (already cited in the introduction of this paper) with its two variants [17, 18], a kernel-based credal classification rule (KCCR) was proposed to deal with the complex data structure. The CCR computes the similarity between the centers of clusters and data points in the input space using Euclidean distance, contrary to KCCR, which adopts a kernel metric to replace the similarity in CCR.

**Figure 1:**
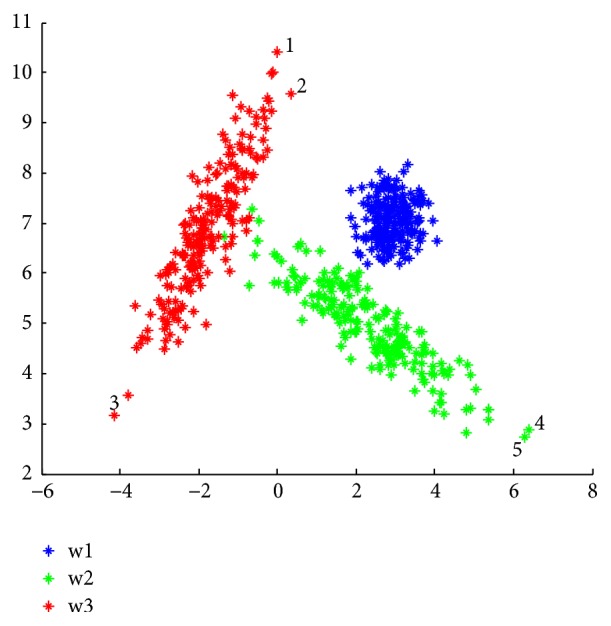
Original dataset.

**Figure 2:**
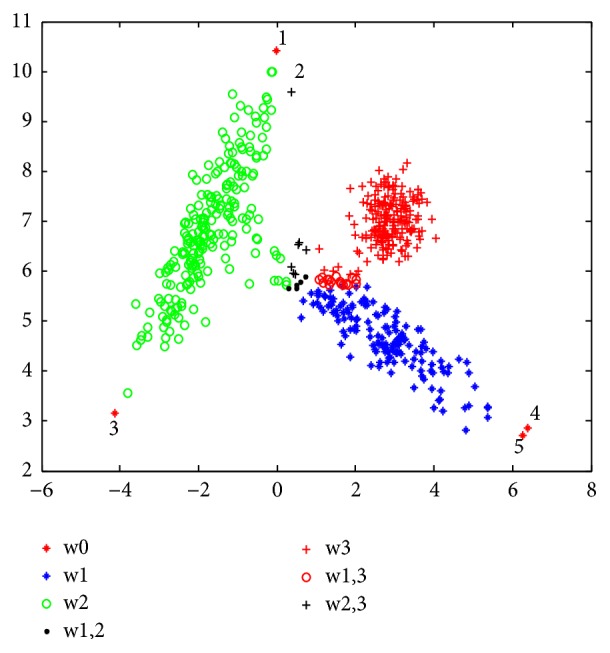
Result with CCR approach by using *γ* = 0.5 (*R*_*e*_ = 3.50%, *R*_*i*2_ = 4.83%).

The limitations of CCR technique are well shown in experimentation 1 (Section 4.1.). In Figure 2, the behavior of the CCR algorithm is presented. It can be inferred that the points whose numbers are “1”, “3”, “4”, and “5” are considered as noise since they are far from the center of the cluster, despite being labelled in a defined cluster. For example, if you take point “1” you see that it belongs to cluster *w*_1_ (see Figure 1), and it is considered as noise in Figure 2. It is noted that points “1”, “3”, “4”, and “5” are well classified by using kernel function in the KCCR and MKCCR algorithm (Figure 4).

### 2.2. Kernel Credal Classification Rule

The kernel credal classification rule (KCCR) as CCR provides a simple method to calculate the masses of beliefs. The KCCR algorithm adds kernel information to the traditional credal classification rules algorithm and it overcomes their disadvantages.

The kernel-based methods involve performing an arbitrary nonlinear mapping from the original dimensional feature space to a higher dimensionality space (also called kernel space). The purpose of using higher dimensions is to be able to apply a linear classifier in the kernel space while the original problem in the feature space could be highly nonlinear and nonseparable linearly.

In order to study the distribution of the data points in a high-dimensional feature space a KCCR approach has been developed [22, 23]. It mainly consists of two steps. Firstly, we determine the center of the specific and metaclusters. Secondly, we construct the BBAs based on the distance between the object and cluster centers.

Let us consider a nonlinear map as Φ : *x* → Φ(*x*) ∈ *F*, where *x* ∈ *X*. *X* indicates the input data space (*ℝ*^*n*^) and *F* the transformed feature space with higher dimension. This nonlinear mapping is defined such that Φ(*x*)^*t*^Φ(*y*) = *k*(*x*, *y*), ∀*x*, *y* ∈ *ℝ*^*n*^, which is called kernel trick [27]. In kernel algorithms, the main idea is that the nonlinear mapping does not have to be explicitly specified.

When Φ is the identity, the function *k* is symmetric, continuous, and positive-definite, so it constitutes a proper Mercer kernel [28]. Polynomial, hypertangent, and Gaussian kernels are examples of Mercer kernel defined, respectively, as follows [29, 30]: *k*(*x*, *y*) = (〈*x*, *y*〉 + 1)^*d*^, *d* ∈ *ℕ*, *k*(*x*, *y*) = 1 − tanh⁡(−‖*x* − *y*‖^2^/*h*^2^), *k*(*x*, *y*) = *e*(−‖*x* − *y*‖^2^/*h*^2^), *h* ∈ *ℝ*^+^.

To obtain the center of each specific cluster, we can use the centers produced by FCM or ECM, or the average of training data.

We consider a metacluster denoted by *U* and *c*_*U*_ is the center of this metacluster. For this the following conditions must be satisfied:

(6) $$\begin{array}{c} \left\|\;c_{U}^{\phi }-c_{i}^{\phi }\;\right\|=\left\|\;c_{U}^{\phi }-c_{j}^{\phi }\;\right\|,\;\forall w_{i},w_{j}\in U,\;\;i\neq j \end{array}$$

and it can be proven that ‖.‖ defined in (6) is a metric in the original space in case that *k*(*x*, *y*) is taken as the Gaussian kernel function, which measures the similarity between the center of specific cluster *c*_*i*_^*ϕ*^ and center of metacluster *c*_*U*_^*ϕ*^.

After determination of the centers of specific and metaclusters, now we construct the BBAs of objects belonging to a specific cluster metacluster or outlier cluster, based on the similarity between the object and the other clusters.

If the object is committed to a specific cluster *w*_*i*_, the initial mass of belief of *x* should be a monotone decreasing function denoted by *f*_1_(.); its expression is as follows:

(7) $$\begin{array}{c} \tilde{m}\left(\;w_{i}\;\right)=f_{1}\left(\;d^{\emptyset }\left(\;x,c_{i}\;\right)\;\right),\;\forall i=1,\ldots ,l \end{array}$$

with

(8) d∅x,ci=Φx−Φci2=Kx,x+Kci,ci−2Kx,ci

where *K*(*x*, *c*_*i*_) = Φ(*x*)^*t*^Φ(*c*_*i*_) and is an inner product kernel function. If we adopt the Gaussian function as a kernel function, i.e.,

(9) $$\begin{array}{c} k\left(\;x,c_{i}\;\right)=e\frac{{-\left\|x-c_{i}\right\|}^{2}}{h^{2}} \end{array}$$

where *h* represent the hyperparameter of the kernel, then *k*(*x*, *x*) = 1.

Hence,

(10) $$\begin{array}{c} d^{\emptyset }\left(\;x,c_{i}\;\right)=2-2K\left(\;x,c_{i}\;\right) \end{array}$$

The Gaussian kernel is the most used in learning algorithms [31] because it contains only one parameter to set and often gives good results.

The determination of the mass of belief of object on metacluster *U* should be done by both the distances to the centers of the specific clusters involved in the metacluster and the distance to the center of this metacluster. Therefore, the mass of belief for *x* on the metacluster *U* should be a function denoted by *f*_2_(.) of both the distance to each center of the specific cluster involved in *U* and distance to center of metacluster *c*_*U*_^*ϕ*^.

(11) $$\begin{array}{c} \tilde{m}\left(\;U\;\right)=f_{2}\left(\;\Delta^{\phi }\left(\;x,c_{U}\;\right)\;\right) \end{array}$$

where

(12) $$\begin{array}{c} \Delta^{\phi }\left(\;x,c_{U}\;\right)≜\frac{1}{\left|U\right|+\gamma }.\left[\;\underset{w_{i}\in U}{\sum }d^{\emptyset^{\beta }}\left(\;x,c_{i}\;\right)+\gamma d^{\emptyset^{\beta }}\left(\;x,c_{U}\;\right)\;\right]. \end{array}$$

In this work as CCR approach, we use the exponential decreasing functions. So, the mass of belief for specific and metaclusters is given by the following:

(13) m~wi=e−λd∅βx,ci

(14) m~U=e−λΔϕx,cU

We note that *λ* is a parameter, which can be determined by the average distance between each pair of centers of the specific clusters.

(15) $$\begin{array}{c} \lambda =\frac{1}{\left(1/2\right)\left|U\right|\left(\left|U\right|-1\right)}.\overset{n}{\underset{i=1}{\sum }}\;\underset{j>i}{\sum }d^{\emptyset^{\beta }}\left(\;c_{i},c_{j}\;\right) \end{array}$$

The mass of belief of the outlier cluster for *x* can be written as

(16) $$\begin{array}{c} \tilde{m}\left(\;w_{0}\;\right)=e^{-\lambda \delta } \end{array}$$

where

(17) $$\begin{array}{c} \delta =\eta \times \arg\left[\;{max}_{i,j}\left(\;d^{\emptyset^{\beta }}\left(\;c_{i},c_{j}\;\right)\;\right)\;\right] \end{array}$$

is a parameter which controls the points belonging to the outlier cluster.

In the next section, the proposed Mahalanobis kernel credal classification rule is detailed. MKCCR is proposed to deal with the limitations of KCCR approach. The example mentioned in experiment 1 shows the limitations of KCCR in comparison with MKCCR. By moving to the kernel space, we can solve the problem of outliers because any aberrant points have been detected in KCCR and MKCCR. But KCCR produces a lot of inaccurate data.

## 3. Mahalanobis Kernel Credal Classification Rule (MKCCR)

The performance of the Euclidean distance is clearly distinguished when the shape of the data is hyperspherical. So, another metric can be used to deal with non-hyperspherical data: Mahalanobis distance can be an adequate choice. In [32], an alternative kernel based on the Mahalanobis distance has been proposed for classification by introducing a symmetric positive defined matrix which allows the discovery of clusters with non-hyperspherical forms. The Mahalanobis distance between two samples can be written as

(18) $$\begin{array}{c} d_{m}\left(\;x,y\;\right)={\left(\;x-y\;\right)}^{T}\Sigma^{-1}\left(\;x-y\;\right) \end{array}$$

where Σ represents the covariance matrix of the set of samples or only the class samples considered. If the covariance matrix is ill-conditioned and/or ill-posed, it means that the presence of small errors in the data distribution or the eigenvalues of the Σ matrix is zero or close to zero. Therefore, its inverse can pose major problems, hence the need to regularize the covariance matrix. In the next paragraph, we describe the regularization process of Mahalanobis distance.

### 3.1. Regularization of Covariance Matrix

The Mahalanobis distance in the characteristic space induced by the kernel can be formulated in terms of evaluation of the kernel, as we explain in the following. We present the regularization formula of the covariance matrix used in the Mahalanobis distance and the final formula of the kernel including this regularized matrix. The covariance matrix can be decomposed according to the spectral theorem as follows:

(19) $$\begin{array}{c} \Sigma =V\Delta V^{t} \end{array}$$

where Δ and *V* are, respectively, the diagonal matrix of the eigenvalues and the orthonormal matrix composed of the eigenvectors.

According to the work proposed by Fauvel in [33], the new formula of the inverse regularized covariance matrix is given by the following:

(20) $$\begin{array}{c} \Sigma^{-1}=V\Lambda \left(\;\tau ,p\;\right)V^{t} \end{array}$$

with

(21) $$\begin{array}{c} \Lambda \left(\;\tau ,p\;\right)=diag\left[\;\frac{1}{\delta_{1}+\tau },\ldots ,\frac{1}{\delta_{p}+\tau },0,\ldots ,0\;\right] \end{array}$$

The regularization parameter *τ* is set to a very small value. Relying on the literature, the value of this parameter is set in *τ* = 0.01 in our experiment.

In [34], the parameter *p* is chosen by using a model generated from Probabilistic Principal Component Analysis (PPCA), but in our work we keep *p* = *d*, where *d* is the data dimension, because our goal is not to reduce the number of dimensions. But we actually want to keep the number of variables that compose the dataset.

### 3.2. Mahalanobis Kernel Credal Classification with Regularization

In this section the Mahalanobis kernel credal classification algorithm is presented in detail. As mentioned in (18), we must calculate the inverse of Σ. For this, the inverse of covariance matrix can be written as follows:

(22) $$\begin{array}{c} \Sigma^{-1}=V\Delta^{-1}V^{t} \end{array}$$

By replacing the Euclidean distance in the Gaussian kernel in (9) with that of Mahalanobis, the Mahalanobis based kernel is defined from the definition of Mahalanobis metric cited in (18). We obtain the following:

(23) $$\begin{array}{c} k\left(\;x,y\;\right)=\exp\left(\;-\frac{{\left(x-y\right)}^{T}V\Lambda \left(\tau ,p\right)V^{t}\left(x-y\right)}{2h^{2}}\;\right) \end{array}$$

In this part, the credal classification rule based on regularized Mahalanobis kernel (MKCCR) is introduced to overcome the limitations of the use of the Euclidean distance. The algorithm MKCCR provides a simple method to calculate the masses of beliefs for singletons clusters, metaclusters, and noise cluster.

The MKCCR as KCCR is an algorithm based on two steps. The first determines the centers of each specific and metacluster, under the condition mentioned in (6). In the second step, the masses of belief of objects belonging to a specific cluster, metacluster, or outlier cluster, based on the similarity between the object and the other clusters will be constructed using (13), (14), and (16). In the first step, an initialization of the cluster centers must be performed. As already mentioned in the first section, there are different methods to initialize the centers; in our work the FCM algorithm was used for this purpose. But it should be noted that this initialization affects the obtained result.  Algorithm 1 summarizes the MKCCR approach.

The performance of MKCCR approach was clearly justified in the dataset used in experiment 1. Clustering results are better compared to those obtained with CCR and KCCR methods, and clusters are distributed with a minimal error rate.

## 4. Experiments Results

In this section, results obtained on simulated and real data sets are presented to show the robustness of MKCCR approach. The hyperparameter *h* of the Gaussian kernel has been chosen according to the tested data.

**The parameters of the algorithm: **before running the MKCCR algorithm, the parameter values must be set. Parameters *β*, *γ*, and *η* have the same meanings as CCR and *h* is the bandwidth of the Gaussian kernel used in the KCCR approach. The value of *β* can be set to be *β* = 2 in all experiments which is a usual choice. The parameter *γ* is used to control the number of objects in the metaclusters; in our experiments the parameter is taken between [0.5,3]. The parameter *η* is associated with the outlier threshold. The bigger *η* gives smaller number of outliers, and it is generally recommended to take *η* ∈ [0.5,2]. The parameter *h* is determined empirically according to the data distribution, so as to have good results.

To compare the clustering results provided by the clustering algorithms, we suppose that the labels are known. In this work, we use two measures usually applicable with credal classification. The first is the classification error (*R*_*e*_) which is calculated by *R*_*e*_ = (*N*_*e*_/*T*)*∗*100%, where *N*_*e*_ is the number of misclassified objects and *T* is the total number of objects under test. The second is the imprecision rate (*R*_ij_) that can be calculated by *R*_*ij*_ = (*N*_*ij*_/*T*)*∗*100%, where *N*_ij_ is the number of objects assigned to the metaclusters with a cardinality value *j*.

### 4.1. Experiment 1: Simulated Data

**Figure 3:**
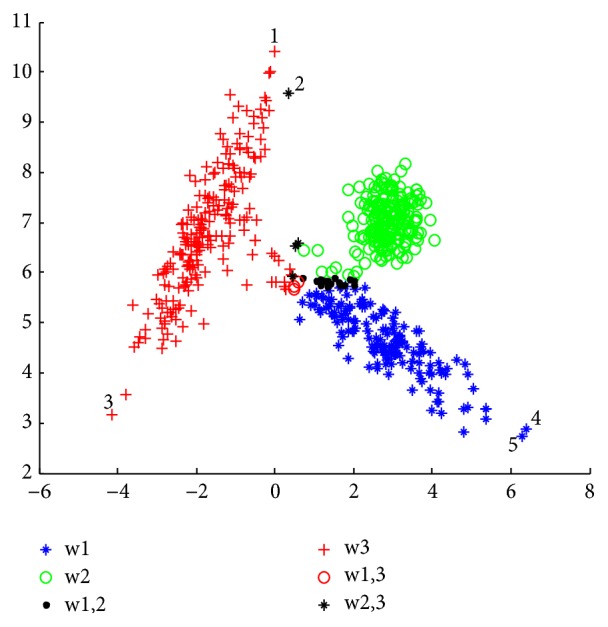
Results with KCCR approach by using *γ* = 0.5 (*R*_*e*_ = 3.50%, *R*_*i*2_ = 4.83%).

**Figure 4:**
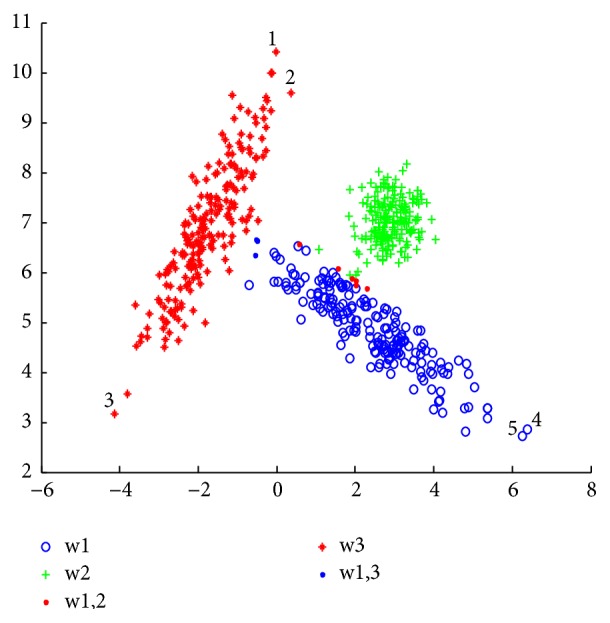
Result with MKCCR approach by using *γ* = 0.5 (*R*_*e*_ = 1.00%, *R*_*i*2_ = 1.83%).

To compare the performance of the Euclidean distance with that of Mahalanobis in the kernel function, a data set of three clusters in 2D is generated as a Gaussian distribution. Each cluster in these data is drawn according to a bivariate normal distribution with mean vector and covariance matrix listed in Table 1. To obtain the data distribution in Figure 1, a rotation matrix with an angle of *π*/3 was applied to the data set. The obtained results by MKCCR algorithm are compared with those of CCR and KCCR algorithms (see Figures 2, 3, and 4).

In Figure 1, *μ*_jk_ represents the components of the mean vector of each cluster and *ρ*_*ik*_ the components of covariance.

In our experiment, we take *α*=2, *h*^2^=20 to show the behavior of the three methods in clustering. The misclassification and imprecision rate obtained by CCR, KCCR, and MKCCR methods are indicated in the subtitle of each figure.

Figure 1 shows the simulated dataset, with the data points for clusters 1, 2, and 3 being, respectively, in blue, green, and red. Figures 2–4 present the results obtained by CCR, KCCR, and MKCCR algorithms. As shown in Figures 2 and 3, the use of Euclidian distance tends to be less efficient contrary to the use of Mahalanobis distance that produces a good performance; see Figure 4. So that, when the covariance of a cluster is greater (for example, the cluster in red color in the original data), the data dispersion is well managed with the Mahalanobis distance. Also, by calculating the error and imprecision rate, we can show that MKCCR is powerful compared to CCR and KCCR.

As shown in Figure 1, some objects that originate from clusters *w*_2_ and *w*_3_ lie in the overlapping zone of the two clusters. These objects are difficult to be correctly committed to a particular cluster *w*_2_ or *w*_3_. In the Figures 2, 3, and 4, these objects are classified by CCR, KCCR, and MKCCR into the metacluster *w*_2_ ∪ *w*_3_, but with different value of imprecision rate. We can observe that MKCCR achieves the lowest imprecision rate of clustering.

The results obtained in Figures 2 and 3 show the wrong behavior of the Euclidean distance when the dispersion of data is not the same for each cluster. In the original data set, we do not find any object which is overlapped between *w*_1_ and *w*_3_, but CCR and KCCR with Euclidean distance provide a metacluster *w*_1_ ∪ *w*_3_ which can be indicated in Figures 2 and 3 by number “2”. By using the MKCCR algorithm with *γ*=0.5, we can find a good compromise between the imprecision rate and error rate in which the imprecision and error rates take 1.83% and 1.00% as values respectively, contrary to CCR and KCCR methods which have a high imprecision rate that reach 4.83% for both methods.

Another disadvantage of CCR approach is that it considers the points numbered “1”,”3”,”4”, and “5”, which are far from the centers, as noisy points as shown in Figure 2 by red star. These points represent 0.67%.

According to the above analysis, it can be concluded that the Mahalanobis distance gives more precision than the Euclidean distance when it is used for a non-hyperspherical data. Therefore, the regularized Mahalanobis distance is introduced to the kernel credal classification rule.

### 4.2. Experiment 2: Real Data Set

The improved KCCR algorithm presented in this paper is applied to the Iris, Wisconsin Diagnostic Breast Cancer (WDBC) and High Time Resolution Universe (HTRU) data sets from the UCI Machine Learning Repository (Iris, WDBC, and HTRU2 datasets can be downloaded from the following address: http://archive.ics.uci.edu/ml), in order to get the performance of our method and to compare this with CCR and KCCR methods.

For the Iris data set, it consists of 150 instances distributed in three clusters. Each cluster contains 50 instances. Each Iris is described by 4 real variables which represent the components of each kind of Iris.

The second database used is Breast Cancer Wisconsin Diagnostic (WDBC). The data set is rich in example, given that the number of patients is equal to 569. It consists of a matrix of 32 columns. The first column represents the patient identifier. This will be ignored in the clustering phase, and in the second column we find the labels M for malignant and B for benign. The distribution of the two clusters is given by 357 benign samples and 212 malignant samples.

The last dataset is named HTRU2: it describes a sample of pulsar candidates collected during the High Time Resolution Universe Survey. Pulsars are a rare type of Neutron star that produce radio emission detectable here on Earth. Each candidate is described by 8 variables. The first four are simple statistics obtained from the integrated pulse profile. The data summary includes 17,898 total examples; it is divided into two classes; the class labels used are 0 (negative) and 1 (positive).

Table 2 shows the results obtained for the used data set. It can be shown that MKCCR provides the best results compared to CCR and KCCR methods. In this experiment the value of the hyperparameter of the kernel is taken according to the type of data. By using Iris dataset, we notice that CCR and KCCR approaches give the same results either for the error rate or for the imprecision rate which are equal to 8.67% and 3.33%, respectively. But the application of our algorithm improves the obtained results in terms of the imprecision rate which is reduced to 2.67% instead of 3.33%.

For the WDBC data set, MKCCR method has lower error rates than KCCR algorithm, which uses Euclidean distance. Using MKCCR, we can see that the obtained results are encouraging, because it can be observed that a compromise has been found between error and imprecision rates by setting *γ*=0.5, contrary to CCR which takes unacceptable behavior for classification, because it generates only noisy points with a rate of 100%. Therefore, CCR is unable to classify this type of data. But for KCCR, the algorithm results in an imprecision rate less than MKCCR which reaches 13.71% but it leaves points belonging to the noise cluster *w*_0_ with a rate of 1.05%. For this, we can conclude that the sum of the elements not classified in this database, by including the points constituting the error and imprecision rate and those belonging to *w*_0_, is greater than that given by MKCCR.

From Table 2, the use of HTRU2 database confirms the advantages of MKCCR. We see that MKCCR algorithm produces the smaller error rate than KCCR method which reaches the value of 15.04% for *γ* = 0.5, instead of using KCCR method that results in 25.53% which is a very high rate. For the classification of this dataset using CCR, the algorithm is unable to detect the two clusters, and it assigns all samples to the total ignorant cluster.

### 4.3. Experiment 3: Dataset

#### 4.3.1. Description of the Dataset

Now, the performance of our contribution will be validated by clustering a set of data from microarray. In this work the Leukemia dataset is considered. Microarray gene expression datasets contain thousands of genes (in Leukemia the number reaches 7,129) but a reduced number of samples are used in our case, 72 including two classes called ALL (Acute Lymphoblastic Leukemia) and AML (Acute Myeloid Leukemia). However, several samples are redundant, which necessitates the application of a filter. For this, different pretreatment methods are used as mentioned in the fourth section in [35]. Among these methods one can find the reliefF filter, the information gain (IG), and the minimum redundancy maximum relevance filter [36, 37].

#### 4.3.2. Discussion of Results

The obtained results for the Leukemia dataset using our clustering approach and other methods are mentioned in Table 3.

To assess our approach, we have chosen different methods for comparison such as ECM, KECM, CCR, and KCCR. The parameters used in the various methods are the same. For this we have taken the hyperparameter of the kernel *h*^2^ = 20, and *γ* = 1. But for the parameter *p* which regularizes the covariance matrix in MKCCR we take it equal to the number of dimensions. The various values of *p* have been taken according to the number of features used. For the ECM and KECM methods, the value *α* = 1 is applied.

By using genes numbers 6041 and 6855, it is noteworthy that the imprecision and error rates are minimal by applying MKCCR, which shows that the algorithm is able to differentiate between the two types of Leukemia Cancer, AML and ALL. Unlike the use of ECM and KECM by adjusting *α* = 1, both approaches reflect the false behavior of the classification since the imprecision rate reaches 15.28% for the KECM algorithm, which shows that a large number of samples remains in uncertainty. The clustering results obtained using CCR and KCCR are less efficient to MKCCR, because we lead to find the best and the lowest error and imprecision rates which are equal to 5.56% and 2.78%, respectively.

Thereafter, the number of variables used was increased to 3 and then to 4 to test the performance of our method. Also, we have varied the value of the parameter *p* according to the number of variables. Here the advantage and the use of Mahalanobis distance are clear.

Regarding the obtained results, we can notice that the MKCCR algorithm performs well on all the genes tested since it introduces both Mahalanobis distance and a regularization term corresponding to the covariance of each cluster into the credal clustering approach which can be suitable to all shapes and sizes of clusters and its robustness to cluster divergence.

## 5. Conclusion

The kernel credal classification with Mahalanobis distance is investigated in this article. MKCCR is an improved version of KCCR method; it is made by replacing the Euclidean distance in the Gaussian kernel with a regularized Mahalanobis distance which take into account the correlation between the variables. The advantage of this contribution is to deal with the clusters which has a non-hyperspherical shapes.

Experimental results using simulated and real datasets have shown that the MKCCR can improve the classification imprecision when compared to a standard Gaussian kernel. All the experimentations show encouraging results of our method compared to CCR and KCCR approaches.

The use of MKCCR for Leukemia dataset has shown good and correct classification behavior towards this type of data, unlike the other methods used. Further experiment is needed to better evaluate the performance and the effectiveness of the proposed method, so our future research will be oriented to many applications such as road safety domain and microarray data analysis.

## Figures and Tables

**Algorithm 1:**
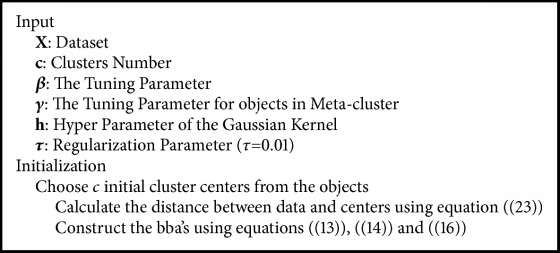
Mahalanobis kernel credal classification rule (MKCCR).

**Table 1:** Mean vector and covariance matrix of the simulated dataset.

	Mean vector	Covariance matrix	Number of elements
Cluster 1	*μ*_11_ = 7,5, *μ*_12_ = 1	*ρ*_11_ = 0,2, *ρ*_12_ = 0, *ρ*_13_ = 0, *ρ*_14_ = 0,2	200

Cluster 2	*μ*_21_ = 5,5, *μ*_22_ = 0,3	*ρ*_11_ = 0,1, *ρ*_12_ = 0, *ρ*_13_ = 0, *ρ*_14_ = 0,3	200

Cluster 3	*μ*_31_ = 5, *μ*_32_ = 5	*ρ*_11_ = 3, *ρ*_12_ = 0, *ρ*_13_ = 0, *ρ*_14_ = 0,1	200

**Table 2:** Error rate and imprecision rate with different methods in %.

	**CCR**			**KCCR**			**MKCCR**		
	**R**_**e**_	**R**_**i**2_	**Noise**	**R**_**e**_	**R**_**i**2_	**Noise**	**R**_**e**_	**R**_**i**2_	**Noise**
**iris** (*h*^2^ = 20)	8.67	3.33	0	8.67	3.33	0	8.00	2.67	0
**wdbc** (*h*^2^ = 250)	NA	NA	100	8.61	13.71	1.05	7.38	15.64	0
**htru2**(*h*^2^ = 100)	NA	NA	100	25.53	7.16	2.95	15.04	12.26	0

**Table 3:** Comparative results for Leukemia dataset.

ID Genes	Approach	*R*_*e*_	*R*_i2_
4847 2020	ECM	2.78%	20.83%
	KECM	2.78%	16.67%
	CCR	4.17%	8.33%
	KCCR	4.17%	8.33%
	MKCCR-reg (p=2)	9.72%	5.56%

6041 6855	ECM	9.72%	12.50%
	KECM	6.94%	15.28%
	CCR	12.50%	4.17%
	KCCR	9.72%	6.94%
	MKCCR-reg (p=2)	5.56%	2.78%

6041 6855 2020	ECM	9.72%	8.33%
	KECM	5.56%	13.89%
	CCR	8.33%	6.94%
	KCCR	6.94%	8.33%
	MKCCR-reg (p=3)	2.78%	1.39%

6041 6855 3252 2020	ECM	5.56%	2.78%
	KECM	5.56%	4.17%
	CCR	6.94%	1.39%
	KCCR	4.17%	4.17%
	MKCCR-reg (p=4)	6.94%	1.39%

## Data Availability

The data used to support the findings of this study are available from the corresponding author upon request.
